# Is the Cyanobacterial Bloom Composition Shifting Due to Climate Forcing or Nutrient Changes? Example of a Shallow Eutrophic Reservoir

**DOI:** 10.3390/toxins13050351

**Published:** 2021-05-13

**Authors:** Morgane Le Moal, Alexandrine Pannard, Luc Brient, Benjamin Richard, Marion Chorin, Emilien Mineaud, Claudia Wiegand

**Affiliations:** 1 ECOBIO (Ecosystems, Biodiversity, Evolution) UMR 6553, University Rennes 1, CNRS, 263 av. du général Leclerc, 35700 Rennes, France; morgane.lemoal@hotmail.fr (M.L.M.); alexandrine.pannard@univ-rennes1.fr (A.P.); luc.brient@univ-rennes1.fr (L.B.); marion.chorin@univ-rennes1.fr (M.C.); emilien.mineaud@gmail.com (E.M.); 2 Département Santé-Environnement, Agence Régionale de Santé de Bretagne, 32 bd de la Résistance, 56000 Vannes, France; Benjamin.RICHARD@ars.sante.fr; 3 Department of Biological Sciences, University of Quebec, C. P. 8888, Succ. Centre-Ville, Montréal, QC H3C 3P8, Canada

**Keywords:** cyanobacteria, eutrophication, long term monitoring, water quality

## Abstract

Cyanobacterial blooms in eutrophic freshwater is a global threat to the functioning of ecosystems, human health and the economy. Parties responsible for the ecosystems and human health increasingly demand reliable predictions of cyanobacterial development to support necessary decisions. Long-term data series help with identifying environmental drivers of cyanobacterial developments in the context of climatic and anthropogenic pressure. Here, we analyzed 13 years of eutrophication and climatic data of a shallow temperate reservoir showing a high interannual variability of cyanobacterial development and composition, which is a less occurring and/or less described phenomenon compared to recurrant monospecific blooms. While between 2007–2012 *Planktothrix agardhii* dominated the cyanobacterial community, it shifted towards *Microcystis* sp. and then *Dolichospermum* sp. afterwards (2013–2019). The shift to *Microcystis* sp. dominance was mainly influenced by generally calmer and warmer conditions. The later shift to *Dolichospermum* sp. was driven by droughts influencing, amongst others, the N-load, as P remained unchanged over the time period. Both, climatic pressure and N-limitation contributed to the high variability of cyanobacterial blooms and may lead to a new equilibrium. The further reduction of P-load in parallel to the decreasing N-load is important to suppress cyanobacterial blooms and ameliorate ecosystem health.

## 1. Introduction

Recurrent and persistent mass developments (blooms) of cyanobacteria are one of the main outcomes of eutrophication of freshwater ecosystems, that is the natural increase in organic matter production and accumulation in an aquatic ecosystem, accelerated during the last decades by human activities [1,2,3,4]. Natural control of cyanobacterial blooms, e.g., by zooplankton, is limited by their low palatability owed to long filaments or colony formation and their low nutritional value, besides their toxicity [5]. Cyanobacterial dominance thus causes a misbalance and malfunctioning of the aquatic ecosystem by supressing the biodiversity of zooplankton, phytoplankton (via competition) and submerged macropytes (by shading), thereby disturbing trophic chains and fluxes [5,6,7]. The decay of phytoplankton including cyanobacteria consumes oxygen, affecting organisms in the water column and ultimately at the sediment. Bottom oxygen depletion limits phosphorus (P) fixation and enhances its release, thus accelerates eutrophication [8]. 

As various cyanobacteria produce bioactive or toxic metabolites, their mass development threatens organisms in the water body or depending on it [9,10,11]. To ensure human safety, many countries use thresholds of cyanobacterial cell densities combined with toxin concentration to restrict access for recreational activities, and the WHO has established guidelines of 1 µg L^−1^ of total microcystins in drinking water [12,13]. Managers and actors responsible for ecosystem and human health increasingly demand reliable predictions of seasonal cyanobacterial development as those regulations may cause financial consequences, including losses in income via recreational activities or increasing costs for drinking water purification. The different cyanobacteria genera comprise distinctive capabilities to form blooms or to produce specific toxins [6], which underlines the necessity to predict bloom formation, composition, duration and heterogeneity in a given water body. 

Despite the urge to return or shift towards the dominance of eukaryotic phytoplankton, this remains difficult, as several physiological mechanisms enable cyanobacteria to outperform eukaryotic phytoplankton. Cyanobacteria grow better in low-light conditions, which occur either during mixing events or due to high phytoplankton biomass, moreover some cyanobacterial species possess gas-vesicles enabling them to adjust their light requirements via buoyancy in the water column [14]. They often benefit from faster uptake kinetics for CO_2_, a higher temperature optimum and lower N concentrations [14]. Additionally, several species are able to directly use dinitrogen as an N-source [8]. All of these features lead to a higher growth rate during summer, by which cyanobacteria can outcompete eukaryotic phytoplankton, leading to recurrent bloom situations. 

Eutrophication is the main driver influencing cyanobacteria blooms, but temperature intensifies bloom frequency, duration and intensity [15]. Shallow lowland lakes suffer more from cyanobacteria because here nutrient loadings meet higher temperatures, and P is easily resuspended from the sediment due to their often polymictic character [6,16]. This applies even more to shallow reservoirs, which continuously receive nutrients (P, N) from the inflowing rivers, charging their sediments as they act as nutrient trap. If not reduced, eutrophication in combination with climate change will thus in future increase cyanobacterial blooms [6,17]. 

Due to the interaction of the driving factors and the differences from one water body to another, it remains difficult to foresee the development and density of a phytoplankton bloom, and particularly its species composition. Long-term data series help identify environmental drivers of cyanobacterial blooms’ occurrence and composition in the context of climatic and anthropogenic pressure and improve predictions for managers. Long term studies previously illustrated the trajectories of eutrophication. This knowledge was applied to reduce point source P pollution that led to a gradual decrease of phytoplanktonic biomass and/or changes in its composition towards eukaryotic species, as demonstrated for eight lakes or reservoirs of many examples by Fastner et al. [18]. The intensification of agricultural practices, however, increased again N and P flux towards adjacent waters, causing numerous lakes and reservoirs to experience or re-experience cyanobacterial blooms. Nevertheless, where effort has been made to reduce agricultural P and N emissions to a lake, it was followed by the amelioration of its water quality [19,20]. Long term (37 years) surveys showed changes in the cyanobacterial community composition towards diazotrophic species as consequence of N decrease while P remained constant, [21], whereas, in another lake, reduction in both N and P decreased also the proportion of N_2_ fixers in the phytoplankton community [20]. 

In accordance with their habitat characteristics in terms of depth, nutrient load, temperature, etc, the same species will dominate recurrent blooms in most lakes. *Microcystis sp.* continuously dominates for example Lake Taihu, China [22,23] or the Aguieira reservoir in Portugal [24], while *Planktothrix agardhii* perennially dominates shallow eutrophic water bodies in lowland areas of the Netherlands and Northern Germany [25,26]. Compared to large monospecific blooms, interannual variation amongst cyanobacterial dominance needs better understanding as it can be a good indicator for changes in climatic and anthropogenic pressure. Interannual variations may indicate a shift towards a new state of the composition of the bloom. 

The investigated shallow reservoir has received its eutrophication with the incoming rivers during the past decades. It shows a high variability of cyanobacterial development between years, for which the driving factors are not yet identified. Aims were therefore 1) to characterize the seasonal dynamic of the nutrient pattern as well as the phytoplankton and zooplankton succession over a one-year period, and 2) to identify the driving factors of cyanobacterial development over 13 years of monitoring, testing the hypothesis that nutrients, phyto- and zooplankton in the reservoir differ along the transect from entrance to outlet (H1), and that even in this short time series, climatic forcing changes the dynamics of cyanobacterial blooms (H2).

## 2. Results

### 2.1. Seasonal Cycle

#### 2.1.1. Nutrients’ Concentration

**Figure 1:**
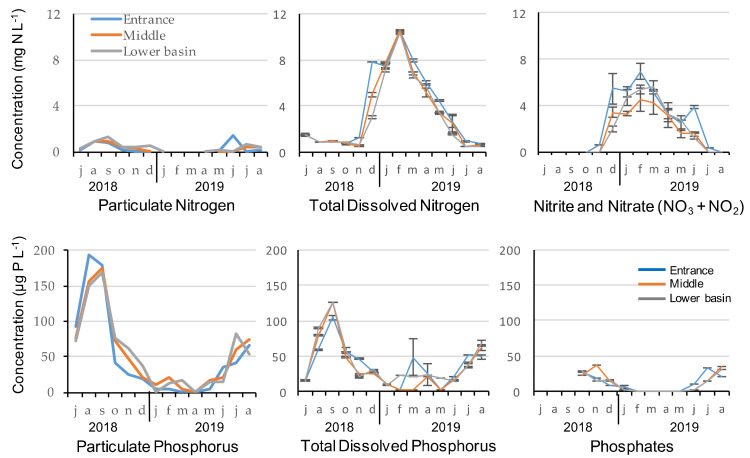
Nutrients concentration at the entrance, middle and in the lower basin of the reservoir during the 2018–2019 seasonal cycle.

Nutrients’ concentrations were very similar at the entrance, in the middle and lower basin of the reservoir during the 2018–2019 seasonal cycle (Figure 1). No significant difference was observed between the three stations in terms of nutrient concentrations but nitrogen and phosphorus concentrations revealed different patterns of seasonal variability. Total dissolved nitrogen and nitrate–nitrite were highly correlated (Spearman correlations, r_S_ > 0.92, *p* < 0.001) and reached maximum concentrations in winter. Particulate N was low, but still corresponded to the particulate P concentrations during that period. Using the theoretical composition of the phytoplankton with the Redfield ratio and the ratios given by Reynolds (2006), we find that 150 µg of P corresponds to 1.05 mg N/L (mass ratio of 47:7:1 for C:N:P) and are therefore consistent with particulate N concentrations of 1.25 mg N/L. During periods of high concentrations in N, dissolved nitrogen reached almost 100% of the total N from January to May, as particulate N remained below LOQ for several sampling dates. During that period, nitrate and nitrite accounted for 65% of the total dissolved nitrogen.

Particulate phosphorus, total dissolved phosphorus and phosphate maximum concentrations were recorded during summer, and both particulate P and TDP were correlated (Spearman correlations, r_S_ = 0.65, *p* < 0.001). Phosphorus concentrations of all fractions were very low from January to May, but reached almost 180 μg L^−1^ for particulate P and 125 μg L^−1^ for TDP at the end of summer, in September 2018. From June to December, total phosphorus was mainly composed of particulate phosphorus for two thirds and TDP for one third. The dissolved fraction accounted on average for 49 ± 24 %, 40 ± 10 % and 40 ± 13 % at the entrance, middle and in the lower basin of the reservoir, respectively. Among this dissolved fraction, more than half was composed of phosphate (mean = 53 ± 10 %, 59 ± 23 % and 53 ± 12 % at the entrance, middle and in the lower basin of the reservoir, respectively). N/P Redfield ratios were low at the entrance of the reservoir (<10 with mean = 6 ± 4), while there were closer to 16 in the middle (>10 with mean = 12.6 ± 1.6) and the lower basin (>10 with mean = 14.9 ± 3.5; Table 1).

#### 2.1.2. Phytoplankton

**Figure 2:**
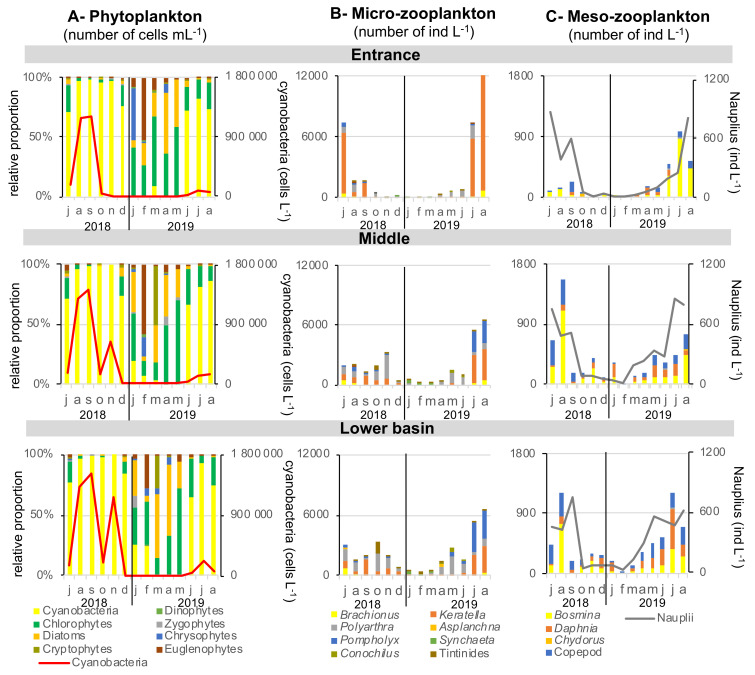
Phytoplankton and zooplankton dynamics over 2018–2019 seasonal cycle at the entrance, middle and in the lower basin of the reservoir. No statistical differences were observed between abundances of the three stations, except for *Polyarthra* (rotifer), copepods and *Daphnia*, for which the entrance present a lower abundance than the lower basin (*p* < 0.05).

The relative proportions of the different groups of phytoplankton and the total abundance of cyanobacteria were very similar at the entrance, middle and in the lower basin, showing no significant difference (Figure 2A). The relative contribution of phytoplankton is calculated from cell number, biovolume being unfortunately unknown, as counting was performed at the genus level. Cyanobacteria dominated planktonic community from June to December 2018 and from June to at least August 2019. There were no cyanobacteria in winter and spring, and their concentrations reached 1,500,000 cells mL^−1^ in late summer (September 2018). A second bloom of cyanobacteria was observed during autumn 2018 in the middle and the lower basin, reaching 600,000 and 1,200,000 cells mL^−1^, respectively, in November. Cyanobacterial density was one order of magnitude lower during summer 2019. Phytoplankton eukaryotic community ranged from 200 cells mL^−1^ in winter to 72,000 cells mL^−1^ in early summer (July 2018). Chlorophytes were the most abundant in this community almost all yearlong, except in spring (March–April) during which diatoms or in february, where euglenophytes were dominating. In January, there was a co-domnance of chlorophytes with Chrysophytes at the entrance but with diatoms in the middle and lower basin.

#### 2.1.3. Zooplankton

Zooplanktonic community abundance and composition was different between the entrance of the reservoir and middle–lower basin, apart from a common pattern of higher density during summers than winter (Figure 2B). Abundances of the rotifer *Polyathra* were significantly higher in the lower basin compared with the entrance (*p* < 0.05). The Cladocera *Daphnia* and copepods had also higher abundances in both the middle and lower basins compared with the entrance (*p* < 0.05). At the seasonal scale, the rotifer species *Keratella* strongly dominated the micro-zooplankton community during both summers at the entrance of the reservoir, reaching 6000 and 12,000 individuals L^−1^ in July 2018 and August 2019, respectively. In the middle and in the lower basin, *Pompholyx* and *Polyarthra* species accompanied *Keratella*, reaching a total maximum concentration of 3000 and 6000 individuals L^−1^ in July 2018 and August 2019, respectively. Concerning meso-zooplankton, the Cladoceran *Bosmina* genus dominated the community in the entire reservoir, together with copepods in a smaller proportion (Figure 2C). The total concentration of meso-zooplankton reached 1200 and 1500 individuals L^−1^ in the middle and the lower basin during summer 2018, while they were 4 to 5 times lower at the entrance of the reservoir (300 individuals L^−1^). During summer 2019, *Daphnia* developed in the middle and lower basin, dominating at 51% the community in July 2019 in the lower basin. Concentration of Nauplii was quite homogenous in the whole reservoir, ranging between few individuals in winter to 800 individuals L^−1^ in summer.

### 2.2. Inter-Annual Variability of Summer Periods

#### 2.2.1. Abiotic Parameters

**Figure 3:**
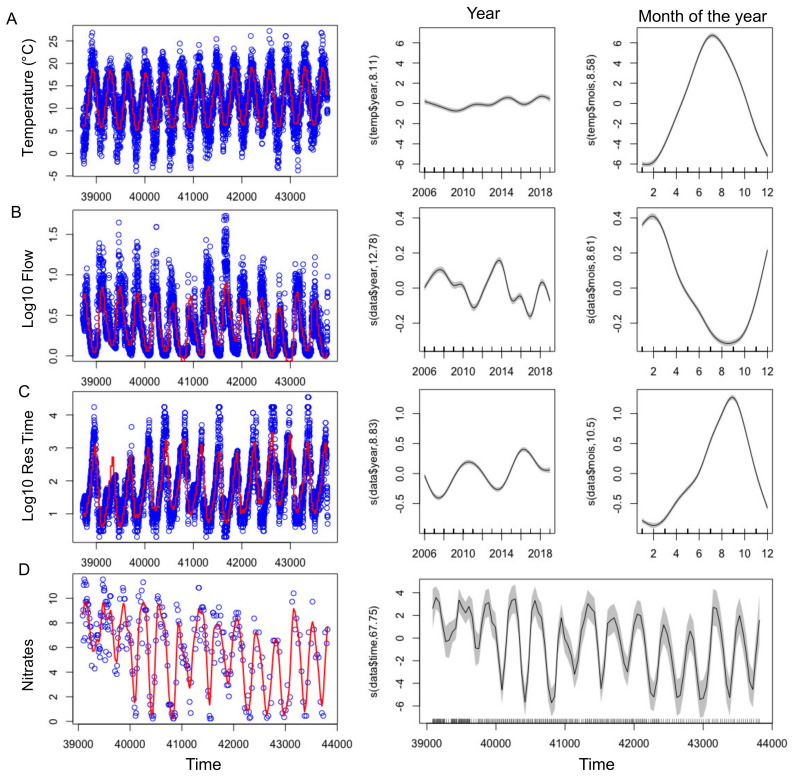
(**left**) Time series of the abiotic factors (blue points), showing the adjusted GAM (Table 3) in red. (**right**) Smooth fit with confidence bands performed with mgcv’s gam function, showing the effect of year and month (or time for Nitrates) on the abiotic factors.

**Figure 4:**
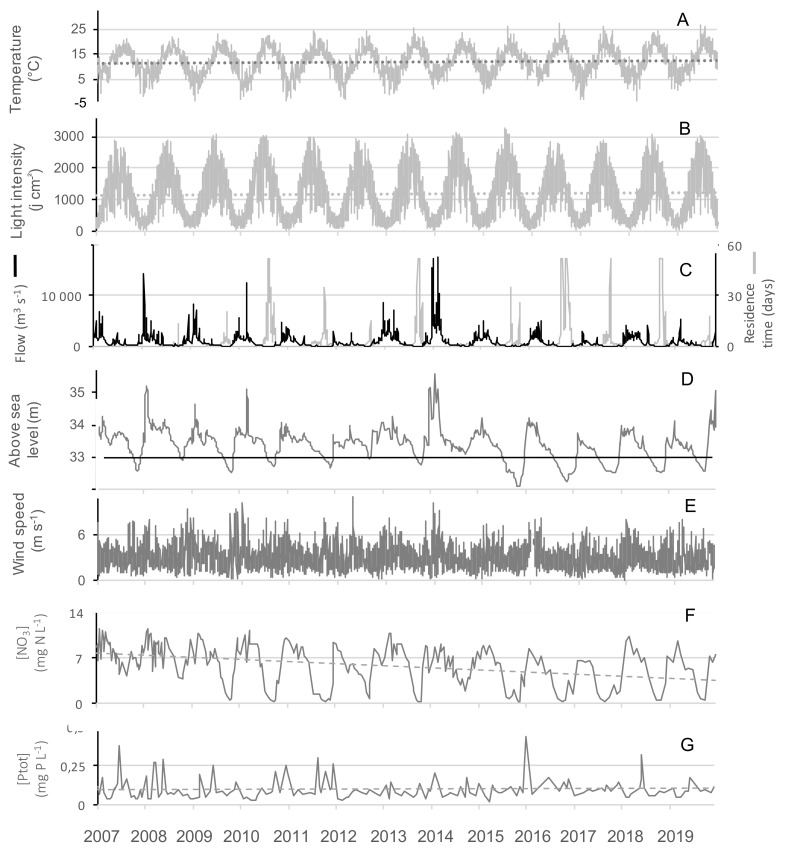
Time series of (**A**) the air temperature, (**B**) the light intensity, (**C**) the residence time and the river inflow, (**D**) the lake water level, (**E**) the mean daily wind speed, (**F**) the nitrates and (**G**) the total phosphorus concentration over 13 years (2007–2019). The light grey areas mark the summer periods. The different dotted lines show tendencies over the 13 years period. The black line in plot D indicates the hight above sea level of the outflow.

With the exception of 2010, summers from 2013-2019 were globally warmer, sunnier and dryer compared to 2007–2012 (Table 2). GAMs were applied successfully to temperature, nitrate concentrations, residence time and flow showing their significant changes over the tested time period (Figure 3, Figure S1 and Table 3). Even during this relatively short time period with respect to long term data, a slight increase of temperature was observed (Figure 3A, Figure 4A) During summers 2010 and 2013–2019, either warm temperature (≥20 days above 20 °C) or high light intensity (≥10 days above 2800 J m^−^^2^) or dry months (less than 1.5 million m^3^ water entering into the reservoir) were recorded (Table 2). Before 2013 (except 2009), a strong and frequent wind was measured in the summers, with at least 19 days of wind with an average daily speed greater than 4 m s^−1^ (Figure 4E, Table 2). After 2013, only 2019 was classified as a windy summer. GAM could not be adjusted on wind due to the high daily variability and explained less than 4% of the deviance (not shown). 

The biggest differences between the years of the study concerned the amount of water entering the reservoir during the hydrological year, ranging from 19 to 155 million m^3^ per year (Table 2). 2012, 2017 and 2019 were the driest hydrological years, having received less than 40 million m^3^. This increased the residence time and lowered the level, and water outflow stopped from July onwards (Figure 3B,C, Figure 4C,D). On the contrary, 2007, 2008 and 2014 presented high flow (Figure 3B) all year round (Table 2).

#### 2.2.2. Nutrients

A strong seasonal pattern was observed for nitrates (Figure 3E and Figure 4F), with maximum concentrations in winter (11.5 mg N-NO_3_^−^ L^−1^) and minimum ones in summer. Owing to the low data points (n = 283), GAM was not able to discouple the seasonal pattern from the interannual one, and we smooth only one independent variable, the time (Figure 3D). Nitrate concentrations slightly decreased over the studied period, and since 2009 concentrations were at the LOD at the end of summer periods, except in 2014 (Figure 3D and Figure 4F). Contrastingly, no seasonal pattern nor long-term tendency was observed for total phosphorus for the 2007–2019 period (Figure 4G). Total phosphorus concentration remained high with on average 100 ± 67 µg P-PO_4_^3^^−^ L^−1^ over the period studied.

#### 2.2.3. Cyanobacterial Blooms 

**Figure 5:**
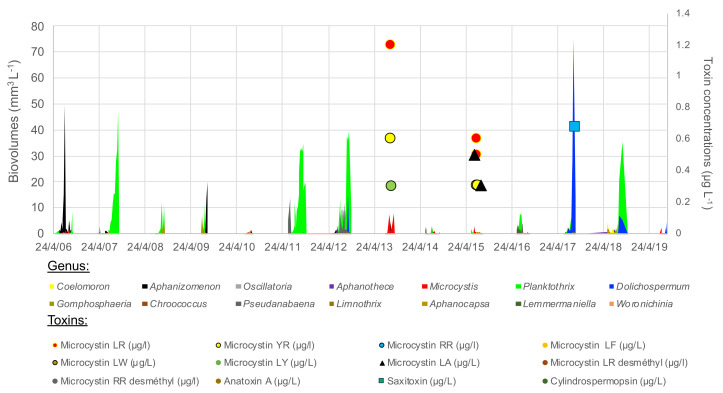
Cyanobacterial genus (in biovolumes, areas) and toxins’ concentrations (points) over 2007–2019 summer periods in the lower basin of the reservoir.

The cyanobacterial community varied strongly interannually, even including years without blooms, 2010, 2014 and 2015 (Figure 5). *Planktothrix agardhii* was the most abundant species, dominating 6 of the 13 monitored years and reaching a biomass of at least 35–55 mm^3^ L^−1^ in 2007, 2011, 2012 and 2018. *Microcystis* (mainly *M. aeruginosa*) dominated in low densities in 2013, 2015 and early 2019, with associated detection of microcystin in low concentration in 2013 and 2015 (Figure 5, Table S1). *Microcystis* and microcystin (-LR, -RR, -YR, -LA, but not the demethylated congeners) were also detected in low density in 2018, a *Planktothrix* dominated year. *Dolichospermum* (mainly *spiroides*) dominated in huge densities in 2017 (75 mm^3^ L^−1^), and was present to a lesser extent in 2012, 2018 and 2019. Saxitoxin was detected in low concentration in 2017 and 2018. Anatoxin-a and cylindrospermopsin remained below LOD. *Aphanizomenon* dominated the cyanobacterial community in 2009. *Aphanothece* and *Aphanocapsa* (picocyanobacteria) dominated the cyanobacterial community in 2010 despite their very small biovolume per cell. All these species were present during summer 2014, but none dominated or bloomed.

#### 2.2.4. Coupling Blooming Species with Environmental Parameters 

**Figure 6:**
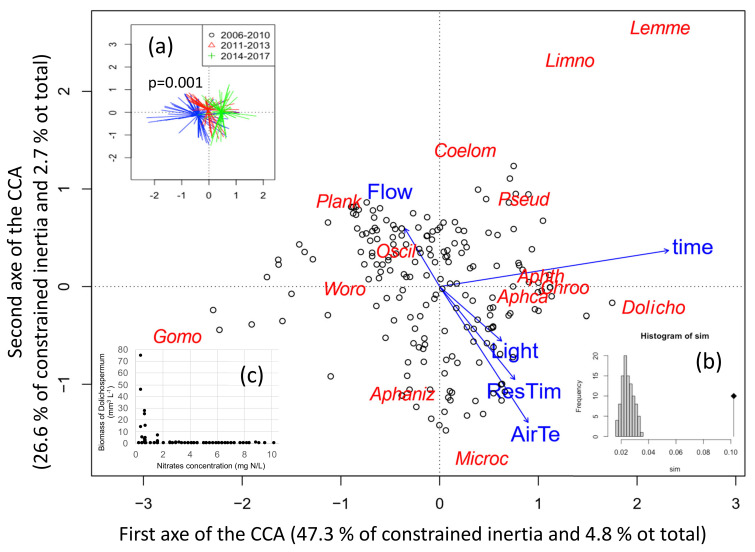
Distance biplot of the Canonical Correspondence Analysis (CCA) linking cyanobacterial biovolumes to environmental parameters measured during the six previous days. Data from summer periods between 2006 and 2017 were used. (**a**) Significant change of cyanobacteria composition with time was tested by permutation test (function envfit). (**b**) Histogramm of the permutation test for the ordination model. (**c**) Biomass of *Dolichospermum* depending on nitrates concentration, showing a threshold effect. ResTim: water residence time, AirTe: mean air temperature, Light: mean daily global radiation, Flow: mean river inflow, and time: sampling dates as open circles. The less significant environmental variables (rainfall, wind in intensity and variability) were eliminated from the analysis based on Akaike Information Criterion (AIC). Cyanobacteria genus by alphabetic order are: Aphaniz: *Aphanizomenon*; Aphca: *Aphanocpasa, Aphth: Ahanothece,* Chroo: *Chroococcus,* Coelom: *Coelomoron,* Dolicho: *Dolichospermum*; Gomo: *Gomphosphaeria*, Lemne: *Lemmermaniella*, Limno: *Limnothrix*, Microc: *Microcystis*; Oscill: *Oscillatoria*, Plank: *Planktothrix agardhii*, Pseud: *Pseudanabaena*, Woro: *Woronichinia.*

To link the cyanobacteria species composition depending on time and environmental parameters, a canonical correspondence analysis has been performed on 195 sampling dates performed during summer. The final CCA included four environmental variables and time, which explain 10.2% of the total variability in the species composition (Figure 6). The CCA discriminated the 12 cyanobacteria species of which 6 formed a bloom. The two first axes of the analysis presented here represent 7.5% of the total variability in species blooming (total inertia) and 73.9% of the constrained inertia. The first axis correlated with time (r = 0.92), hence when samples are grouped by sampling dates, old dates appear on the left while recent ones are on the right (Figure 6a). The first axis correlated moreover to air temperature (r = 0.35), residence time (r = 0.3) and light (r = 0.25) (all towards the positive side). 

The first axis is also explained by *Planktothrix* (23.7%) and *Gomphosphaeria* (20.3%) on the negative side and *Dolichospermum* (14.2%), *Aphanothece* (13.9%), *Lemmermaniella* (8.3%) and *Aphanocapsa* (5.6%) on the positive side. 

The second axis negatively correlates with air temperature (r=-0.73), residence time (r = −0.50) and light (r = −0.29), and positively with flow (r = 0.31) (Figure 6). Sampling dates with low flows thus coincide with high temperature, residence time and light. Air temperature and residence time thus contribute to both axes. The second axis is explained by *Microcystis* (28.2%) and *Aphanizomenon* (24.3%) on the negative side (bottom part – low flow), and by *Planktothrix* (18.2%), *Lemmermaniella* (11.7%), *Coelomoron* (7.0%) and *Limnothrix* (3.5%) on the positive side (upper part–high flow). All blooming species thus contributed at least to one of the two axes, while *Planktothrix* contributes negatively to both of them, in opposition with air temperature and residence time.

It should be noticed that nutrients were not included in the CCA analyses because they were measured less frequently and not necessarily at the same times than the phytoplankton community, moreover P concentrations did not change during the period of the data series. *Dolichospermum* sp. however seems to benefit below concentrations of 2 mg L^−1^ of nitrogen (Figure 6c): blooms of *Dolichospermum* were indeed only observed at very low nitrate concentrations.

## 3. Discussion

A low spatial variability but a high interannual variability in cyanobacteria biomass and composition have been revealed by this study. The seasonal cycle characterized this shallow reservoir as relatively homogeneous, with a similar evolution of nutrient concentrations and phytoplankton abundances, but different patterns for zooplankton along the increasing distance and depth from the entrance to the lower basin. These findings reject our first hypothesis assuming differeces concerning most parameters, but it could be accepted for the zooplankton dynamics after further investigations.

Maximum concentrations of phosphorus and nitrogen were both high, but with strong opposite seasonal patterns: the highest phosphorus concentrations occurred in summer when nitrogen was the lowest. Phosphorus concentrations of the lake were 3 times higher in the reservoir in summer 2018 compared to stations measured in headwaters of the incoming river Yvel [27]. As both nutrients usually enter during the wet winter months [28], their opposing seasonal pattern could indicate either enhanced uptake or denitrification for N, whereas the P concentrations exceeding those of the incoming water could have been released from the sediments during summer, as known from other lakes [20,29,30]. 

In summer, the Redfield N/P ratios in the total fraction were always below 20, confirming a deficiency of bioavailable N in the water column during this period [31]. This result is in line with an analyse of 369 German lakes concluding that N limitation seems to predominate during summer in shallow polymictic lakes [29].

Phytoplankton and zooplankton succession and densities were similar to many eutrophic water bodies. Total abundances of meso-zooplankton appeared high, but remained in agreement with other shallow eutrophic lakes [32,33,34]. Small zooplanktonic taxa seemed to dominate at the detriment of larger cladoceans, e.g., *Daphnia* sp., as known from eutrophic temperate lakes of both Europe [34] and the USA [35]. As sampling size was low in this study, more research focusing on zooplankton dynamics in that reservoir would be beneficial.

An interesting element in the seasonal cycle was the interannual variability between the two summers: while the bloom of cyanobacteria reached an exceptional high value of 1.5 million cells mL^−1^ in 2018, exceeding the limit allowing bathing by a factor of 15 in France and other countries [36], it was one order of magnitude inferior in 2019. At the same time, in the lower basin the zooplanktonic community switched from a dominance of *Polyarthra* and *Bosmina* during 2018 to a dominance of *Keratella, Pompholyx* and *Daphnia* in 2019, which could have been provoked by decreasing cyanobacteria in the phytoplankton community. Some zooplankton taxa such as the raptorial rotifer *Polyarthra*, or to a lesser extent the cladocerans *Bosmina*, are highly selective feeders avoiding cyanobacteria compared to filter feeders like *Daphnia* that thrive better in the absence of cyanobacteria [37,38,39,40]. A zooplankton community can also be top-down controlled by zooplanktivorous fish preferring large zooplankton such as *Daphnia* [41]. Despite the Lac-au-Duc reservoir being a frequented fishing site, the lack of published data on the fish compartment does not allow us to discuss their potential role in structuring the planktonic community.

The contrast of cyanobacteria abundance between the summer of 2018 and 2019 is in the range of the interannual variability of blooms’ intensity and species composition in the reservoir Lac-au-Duc observed during 13 years. Within the 2007–2019 period, densities of 40 to 80 mm^3^ L^−1^ were reached during five summers, while in the other eight summers it ranged at maximum from 1 to 20 mm^3^ L^−1^. 

Towards the end of the time series (2018–2019), a dominance or co-dominance of *Dolichospermum* replaced the dominance of *Planktothrix agardhii* until 2013 together with a co-occurance of *Microcystis* (mainly *aeruginosa*) in low densities in 2013, 2015 and early 2019. This shift in 2013 of cyanobacterial dominance seems to correlate to dryer and warmer summers connected to an increase of water residence time, a slight decrease of wind and a decrease of N sources, which in total confirms our second hypothesis (climate forces as main drivers). Before 2013, with the exception of 2009, summers were indeed characterized by more windy days. Blooms of *Planktothrix agardhii* dominated during that first period, as this species tolerates low average insolation in turbid waters of polymictic lakes [26,41]. *P. agardhii* also grows best at temperatures between 10–20 °C [42], and may become disadvantaged by warmer temperatures. Additionally, at the regional scale in Brittany, *P. agardhii* was the dominant taxa of the freshwater cyanobacterial community between 2004 and 2011 [43].

Surprisingly, none of the measured microcystin congeners was detected during *Planktothrix agardhii* blooms despite *Planktothrix* sp. being able to form toxic blooms in temperate freshwater ecosystems [44,45]. Variation of toxin production can be explained by (i) presence/absence of *mcy* genes necessary for their synthesis and (ii) individual variation within *mcy* genotypes with inactivation or regulation at the level of genes expression [46,47]. Natural *Planktothrix agardhii* blooming populations can be composed of microcystin producing (*mcy*) and non-producing (non-*mcy*) genotypes, and their proportion can vary considerably [47,48,49]. Moreover, it was demonstrated on non-mcy strains of *P. agardhii* isolated from nine European freshwater bodies to have lost more than 90% of their *mcy* genes during evolutionary processes [50]. Based on the analyse of 138 *Planktothrix* strains from three continents, the variable spatial distribution of *mcy* and non-*mcy* genotypes was suggested to depend on ecophysiological adaptation [51]. In laboratory experiments, non-mcy strains of *P. agardhii* seem to have better fitness than *mcy* strains under non-limiting conditions [52]. Similar results were obtained for *Microcystis*: non-*mcy* strains dominated under optimal growth conditions [53]. The authors of these studies then hypothesized that when cyanobacteria grow under favourable environmental conditions, the cost of producing microcystins becomes too high compared to the advantages it can bring [52]. Based on these results, it is tempting to hypothesize that non-limiting growth conditions concerning P and N may have favoured the selection of non-*mcy Planktothrix agardhii* strains in the Lac-au-Duc reservoir. It would be interesting to verify the presence of *mcy* genes, especially since microcystin detections were demonstrated to negatively correlate to *Planktothrix* biovolume in several Brittany lakes [43], suggesting that non-*mcy P. agardhii* strains are prevalent at the regional scale. From the composition of the cyanobacterial bloom, also other toxins, such as anatoxin-a and cylindrospermopsin could have been expected [44,54], they were however never detected during the monitoring between 2007–2019. 

Since summer 2013, *Microcystis* and *Dolichospermum* became dominant or codominant, related to generally calmer conditions. Both genera are known to benefit greatly from water column stability as they can regulate their buoyancy according to their requirements in the illuminated area of stable lakes [55,56,57]. *Microcystis* occurrence was moreover strongly related to light intensity and temperature, which enhances the stabilization of the water column and favoured the development and persistence of *Microcystis* blooms in other lakes as well [16,58]. Temperature also benefits directly cyanobacterial development through their growth rate [59], and has been the most important factor driving the development of *Microcystis* sp. as analysed in more than 1000 lakes [60]. At the regional scale, light intensity was identified as the main climatic driver for *Microcystis* sp. [43]. Despite dominant in 2013 and 2015, *Microcystis* abundances and associated toxin production remained low in Lac-au-Duc compared to other lakes (e.g., [16,24], suggesting that the favourable conditions for its development were not fully met. The optimal growth rate for *Microcystis* is well above 25 °C [59], a temperature rarely reached in Lac-au-Duc or in the surrounding region [43]. We can also hypothesize that *Microcystis* was limited by nitrogen in 2018 and 2019, when the species dominated in early summer but disappeared thereafter. Although *Microcystis* is able to use diverse forms of nitrogen, N availability appears to be an essential element controlling the development of this cyanobacteria and its toxin production [61,62].

This context of N limitation in Lac au Duc could also explain the emerging occurrence of the diazotrophic *Dolichospermum* in recent years, as underlined by the threshold above which its occurance decreases. Nitrate concentrations progressively declined during the last decade in the river entering the reservoir, as in other rivers at the regional scale [63]. Thus, N-fixation ability provides an ecological advantage for this cyanobacterium [41].

Indeed, the first occurrence of *Dolichospermum* in 2012 and its largest bloom in 2017 corresponded to the driest hydrological years of the time series, that is the years with the lowest nitrogen recharge. Drought also seems to have an impact on the hydrological functioning of the reservoir: from 2015 onwards, due to the succession of rather dry hydrological years, the water level remained low, when outflow stopped from beginning of July until autumn. Water movements were doubtless strongly reduced and this phenomenon was probably exacerbated during dry summers such as in 2017 and 2018, when the inflow of water ceased already in early summer. Air temperature, residence time and light contributed to both axis of the CCA, therefore jointly with time on the first axis, indicating that both parameters tend to increase over time. The occurrence of *Dolichospermum* was seems partly related to a long water residence time producing a favourable environment for these buoyant cyanobacteria. These results are in accordance with those of Hayes et al. (2015) who found evidence in 42 lakes from agricultural watersheds that droughts strongly influence the system towards N limitation and induce the development of diazotrophic cyanobacteria.

We hypothesize that the lake is in the progress to shift to a new equilibrium, dominated by N_2_ fixing cyanobacteria. This is potentially connected to toxin production, but evidence for a causal link between reduced N loading and diazotrophic cyanobacteria such as *Dolichospermum* abundance or biovolume is mixed [57]. In addition, evidence is increasing that N_2_ fixation cannot always compensate significantly for the N deficiency, underlying the need to continue reducing emission of both nitrogen and phosphorus in the catchment [20,64,65,66,67]. Thus, a further monitoring of the cyanobacterial community is recommended.

This study provides another proof for the necessity to apply a catchment wide approach to limit P and N entrance into lakes and reservoirs. While reducing N was successful, it remains difficult to reduce non-point source P, despite efforts undertaken by farmers in changing agricultural practices. Moreover, depending on the water-bodies’ bathymetry and the P stocked in the sediment, the time to reach and restore less eutrophe conditions needs to be taken into consideration. Many environmental parameters can be intercorrelated (nutrient availability, residence time, surface water temperature, stratification, etc.), making it impossible to separate the individual effect of these factors on a community. The complementary approach to such environmental surveys is to use very long term data, a multiple sites approach or mesocosms experiments. The intensity (frequency, stations and parameters) of long term sampling campaigns may present shortcomings, as in our case due to the restauration attempts with applications of CuSO_4_ amongst others, thus we were forced to eliminate several of the years, which may have weakened the data set. 

## 4. Conclusions

To conclude, over only 13 years, two major shifts in the cyanobacterial community have been recorded: a first shift in 2013 from a mixing tolerant population of *Planktothrix* to a buoyant species, *Microcystis*, preferring more stable water conditions, influenced by generally calmer and warmer conditions. A second shift from 2017 onwards was driven simultaneously by droughts (representing the biggest change) and reduced N loadings favouring the diazothrophic *Dolichospermum*. Our study also points out that if P reduction is not successful, dominance of *Microcystis* sp. and *Dolichospermum* sp. potentially increase. It is tempting to predict that these buoyant cyanobacteria will replace the *Planktothrix agardhii* population permanently in the context of climate forcing favouring warmer and dryer conditions at the global scale if N and in particular P cannot be reduced below their requirements [45,68]. This could imply many changes in terms of potential toxin production and increased persistance of cyanobacterial populations from year to year with the growing stock of cells in sediment as both *Microcystis* and *Dolichospermum* have dormant cells, while *Dolichospermum* possesses in addition akinetes. 

Changes can also concern curative actions as buoyant cyanobacteria are sensitive to artificial mixing for example, in contrast to *Planktothrix* [69]. Thus, it will be necessary to follow the evolution of future years to confirm or deny the persistent installation of *Microcystis* or *Dolichospermum* in relation to climatic variation.

## 5. Materials and Methods

### 5.1. Study Site

**Figure 7:**
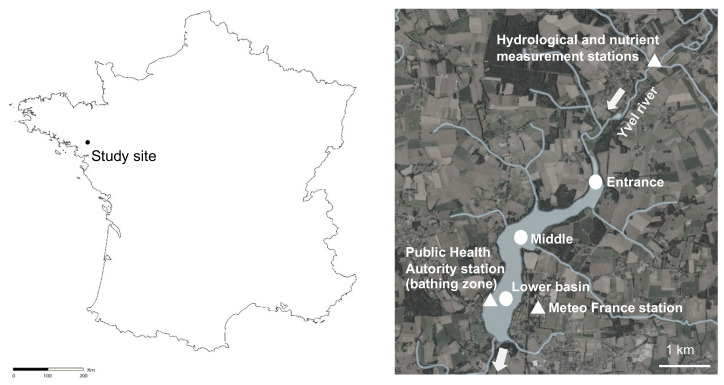
Localization of the Lac-au-Duc in France and stations monitored during the 2018–2019 seasonal cycle (round) for the 13 summer period analyses (triangle). Maps extracted from Geoportail.

The study site, called “Lac au Duc”, is one of the largest shallow water bodies (250 ha, 2.6-m depth in mean) in Brittany (western France, Figure 7). This 3-million m^3^ recreational and drinking water reservoir drains an agricultural catchment (37,000 ha) with the Yvel river as the main tributary. The lake is used for bathing, fishing and nautical activities.

Two types of monitoring data were used in this study: (i) data acquired monthly over a 2018–2019 seasonal cycle at three points in the lake and (ii) data acquired almost weekly over 13 summers, from 2007 to 2019 in a bathing zone. Additional environmental data were collected from governmental and meteorological data bases. 

### 5.2. Seasonal Cycle 2018–2019

#### 5.2.1. Sampling Sites and Sampling 

To examine if nutrients, phyto- and zooplankton were evenly distributed in the reservoir we realized a seasonal cycle. Samples were collected from July 2018 to August 2019 at the entrance, in the middle and in the lower basin of the reservoir (Figure 7) from a boat. Duplicates of five liters of sub-surface water were collected with a 1 m vertical tube sampler at each point for nutrient, phytoplankton and zooplankton analyses. Duplicate samples for dissolved nutrient analyses were filtered on board on sterile Minisart CA 0.45 µm. Nutrient samples were kept at 4 °C until return to the lab, where they were stocked at −20 °C until analysis. Temperature and oxygen were measured along depth profiles with an Idronaut Ocean Seven 316 Plus CTD. 

#### 5.2.2. Nutrients

Particulate phosphorus, total dissolved phosphorus (TDP), particulate nitrogen and total dissolved nitrogen (TDN) were measured by colorimetry after digestion with persulfate according to [70], with a limit of detection of 6 µg P L^−1^ and 50 µg N L^−1^. Orthophosphate (PO_4_^3−^) was analysed by the ammonium molybdate method [71] with a limit of detection of 3 µg P L^−1^. After nitrate (NO_3_^−^) reduction into nitrite (NO_2_^−^) with vanadium chloride, NO_2_^−^ (originally present and reduced nitrates) was measured by colorimetry using sulphanilamide and N-1-naphthylethylenediamine dihydrochloride [72], with a limit of detection of 50 µg N L^−1^. Colorimetric measurements were realised using a Gallery Photometric Analyser Gallery Plus (Thermo Fisher, Saint Herblain, France). 

#### 5.2.3. Phytoplankton 

For the seasonal cycle, microalgae and cyanobacteria composition was determined directly in the fresh sample when possible or were preserved in Lugols solution and stored at 4 °C. Identification were realized to the genus level according to Komárek. Phytoplankton cell counts were carried out with a Nageotte cell according to [73]. For large colonies, the number of cells was estimated using photos. Microscopic photos were used to assess the number of cells in colonies of picocyanobacterial, using Pegasus software. At least, 400 individuals were counted per sample. For both, a light microscope (Olympus BX 50, Rungis, France) was used. Prior to cell counts and identification, if necessary, microalgae and cyanobacteria were concentrated on a 1 μm Poretics polycarbonate membrane filter, thanks to a filtration pump but with low vacuum pressure. Transfer and resuspension of cells in 1 ml of water was done while the membrane was still wet. Intact colonial chlorophytes and cyanobacteria were checked on the microscope to ensure that damage due to the concentration of cells was low. 

#### 5.2.4. Zooplankton 

Zooplankton was concentrated from two liters of water by filtering through a 50-µm net, then narcotized with soda water and preserved in 70% ethanol at 4 °C until counting. For counting, 1 mL of the concentrated sample was distributed on a Sedgewick-Rafter counting chamber. For each sample, a minimum of 400 rotifers were counted and identified at the genus or species level, according to USEPA protocol [74] with a Zeiss microscope, based on the identification manuals for rotifers [75] and cladocerans [76]. In low-abundance samples, the totality of the 1 ml was observed. In addition, crustaceans and cladocerans were counted.

### 5.3. Long Term Series 2007 to 2019

#### 5.3.1. Phytoplankton and Toxins 

Each summer since 2004 the regional public health authorities (French Agence Régionale de Santé, ARS) of Brittany monitor water from a bathing zone located in the lower basin of the reservoir (Figure 7). Depending on the bloom density, samples were collected weekly from June to September, except in 2009 (20/7 to 31/8) and 2010 (24/6 to 30/8). Monitoring stopped in mid-September in 2015–2019, while it continued until end of October in 2011 and 2012. Cyanobacteria abundance and species composition as well as toxin concentrations were analysed by local laboratories. Toxins were analysed with LC-MS/MS [77] after extraction with methanol. Microcystins analysed comprised the congeners microcystin—LR, YR, RR, LF, LW, LY, LA, desmethyl-LR and desmethyl RR with a limit of quantification (LOQ) of 0.3 μg L^−1^. MC-LR-eqivalents were calculated according to Wolf and Frank [78] and Fastner and Humpage [79] with MC-LR (1 per definition), MC-LA, MC-YR, MC-YM (1.0) and MC-RR (0.1). Saxitoxin was determined with an LOQ of 0.6 μg L^−1^, anatoxin LOQ < 0.3 μg L^−1^ and cylindrospermopsin LOQ < 0.6 μg L^−1^. All cyanobacterial cell densities data were converted to biovolume with the use of relevant geometric formulas and data literature [80,81], to account for differences in contribution from larger species and smaller species. 

#### 5.3.2. Abiotic Parameters 

Daily meteorological data (air temperature, global radiation intensity and wind speed) were collected from Météo-France database from 2007 to 2019. Measurements were realised at the Météo-France station of Ploërmel, located 0.5 km from the reservoir (Figure 7). The light energy sensor broke in July 2017, from this date measurements from the Rennes-St-Jacques Météo-France station, located 60 km from the reservoir, were used. The number of days with an air temperature above 20 °C, or with solar radiations greater than 2800 J cm^-2^ were calculated. The water flow as well as nutrient concentrations were measured in the Yvel river, 3 km upstream the entrance of the reservoir (Figure 7). The water flow data were provided for the 2007–2019 period from the national database Hydro France [82] and corresponded to the daily mean values calculated from continuous stage records. From these data, we calculated the residence time of the water in the reservoir, knowing that its volume is approximately 3 million m^3^. To take into account the time needed between climate variation and phytoplankton response, hydrological and meteorological data were averaged over 6 days preceding the sampling date of cyanobacteria. Wind variation completed the average data. Windy summers were identified by the number of days of wind above a threshold of 4 m s^−1^ during a 24 h average following [82]. Monthly data for nitrate and total phosphorus concentrations were provided from the database of the Yvel-Yvet watershed manager (the Syndicat Mixte du Grand Bassin de l’Oust, SMGBO) from 2007 to 2019. By combining these nutrient data with flow data, we calculated the quantities of NO_3_^−^ and Ptot entering the reservoir over the summer period or the complete hydrological year (November to November). The city of Ploërmel also provided reservoir water level data from the drinking water purification station.

#### 5.3.3. Statistical Analyses 

Despite being available, we discarded data from 2002 to 2005 as some copper sulphate treatments had been applied in the whole reservoir up to summer 2005. Treatments with calcium carbonate and hydrogen peroxide also took place, but locally in the bathing area, between 2013 and 2015 and in July 2018, respectively. During these years, except 2018, no significant difference was observed between the bathing area and the rest of the lake. We then chose to keep the data from the long-term monitoring bathing area for 2013–2015, while we used data from outside of the enclosed area only in 2018. Monitoring of nutrient concentrations started in 2007, thus data from 2006 were not used in analyses, leaving an extra year after the copper sulphate treatments.

All statistical analyses were performed in R Studio version 1.2.5019 [83]. In order to link cyanobacterial blooming genera with environmental parameters and characterize the sampling dates, a canonical correspondence analysis (CCA) was performed on Hellinger transformed genera biovolume (response matrix) and centred-reduced environmental parameters (explanatory variables). To reduce the number of explanatory variables, the ordistep function of the vegan package has been used to find the most parsimonious model based on Akaike Information Criterion (AIC). The less significant explanatory variables were eliminated from the CCA, in order to identify only the significant ones. The significance of the final CCA has been tested through a permutation test with the function envfit. A reference distribution under H0 from the data themselves is generated by permutations of rows and columns and by calculating the new percentage of constrained variance (sum of all canonical eigenvalues). The collinearity between explanatory parameters has also been checked.

We used generalized additive models (GAM) for time series of abiotic parameters, to analyze their temporal dynamics according to the season and the year. GAM takes into account the nonlinear response between the dependent variable (the abiotic parameter) and the explanatory variables (time, month of the year and year), using smooth functions called splines. Finally, the final shape of the relationship is determined by the data themselves. The independant variables in the initial model were the month of the year and the year, except for nitrates, for which it was only time (date) due to little data (Table 3). The best model was selected based on the gain in deviance explained (DE) relative to the initial model, while minimizing the Akaike’s information criterion (AIC). Models were adjusted using the R package ‘mgcv’ and the function gam() with the selection of the Restricted Maximum Likelihood (REML) method. The family was Gaussian and the link function was identity. A Log 10 transformation was done on the abiotic param when needed. 

## Figures and Tables

**Table 1:** N/P Redfield ratios of the particulate matter at the entrance, middle and in the lower basin of the reservoir, respectively. N/P Redfield ratios were calculated only for the July–November period, as during the rest of the year, there was no particulate nitrogen and/or phosphorus concentrations were below limit of quantification.

		N/P Ratios of the Particulate Matter		
		Entrance	Middle	Lower Basin
2018	July	5.45	10.16	10.34
	August	10.67	12.99	14.62
	September	10.00	12.43	16.41
	October	7.61	11.62	11.18
	November	1.15	12.23	13.83
2019	July	0.28	15.45	17.80
	August	6.78	13.16	19.92
	Mean ± SD	5.99 ± 4.03	12.58 ± 1.62	14.87 ± 3.46

**Table 2:** Hydrological and meteorological characterisation of summer periods between 2007 and 2019. “Annual river inflow” was measured between November of the previous year and October, while “Summer river inflow” was measured between June and September. Dates of downstream flow stop (out of the lake) are indicated and depends on the water level (overflow). The date of the autumn increase in river inflow is also indicated in the last column.

	Number of Days from June to September			River Inflow in the Reservoir:		Date at Which Downstream Flow is Closed to 0	Date of Autumn Discharge Beginning
	With Water Temperature > 20 °C	With Light > 2800 J cm^−^^2^	With Daily Wind > 4m s^−1^	Annual River Inflow (m^3^)	Summer River Inflow (m^3^)		
2007	2 days	1 days	25 days	104,466,499	14,288,573	outflow all year round	
2008	4 days	6 days	29 days	82,705,190	6,988,378	outflow all year round	
2009	10 days	5 days	11 days	72,962,554	3,228,854	26/08/2009	17/11/2009
2010	19 days	15 days	27 days	74,001,686	900,547	27/07/2010	07/10/2010
2011	11 days	9 days	33 days	53,371,440	879,984	24/09/2011	15/12/2011
2012	14 days	3 days	22 days	30,433,277	3,377,462	14/09/2012	05/10/2012
2013	25 days	10 days	18 days	105,320,477	3,085,603	12/09/2013	06/11/2013
2014	18 days	16 days	7 days	155,305,037	2,709,418	outflow all year	
2015	12 days	18 days	18 days	68,434,762	1,434,931	05/07/2015	23/11/2015
2016	20 days	1 days	7 days	62,350,906	2,336,688	25/07/2016	26/01/2017
2017	23 days	3 days	11 days	19,428,682	1,200,269	03/07/2017	15/12/2017
2018	30 days	8 days	10 days	71,447,098	7,710,250	23/07/2018	07/12/2018
2019	25 days	13 days	29 days	38,914,906	2,677,622	12/07/2019	26/10/2019

**Table 3:** Summary of the GAM results performed on the abiotic parameters, with their smoothing functions. Plots are shown in Figure S1. Adj., adjusted.

Dependant Variable (Number of Data Points)	GAMs Model			Smoothing Functions	Estimated Degrees of Freedom	Fisher Test	*p*-Value
	Adj. R^2^	DE (%)	REML				
Temperature (n = 5019)	0.7	70.1%	12598	S (year)	8.105	12.04	4.92 × 10^−16^
				S (month)	8.584	1292.95	<2 × 10^−16^
Log10 Res Time (n = 5052)	0.719	72%	3360.3	S (year)	8.829	129.8	<2 × 10^−16^
				S (month, k = 12)	10.497	1073.5	<2 × 10^−16^
Nitrates (n = 283)	0.817	86.1%	612.37	S (time)	67.75	15.15	<2 × 10^−16^
Flow (n = 5027)	0.651	65.2%	−970.35	S (year)	12.78	71.01	<2 × 10^−16^
				S (month)	8.61	8.61	<2 × 10^−16^

## Data Availability

Long term data series were obtained from the following sources: Cyanobacteria: ARS Bretagne, France; Temperature, radiation and wind speed: Meteo France, Ploermel, respectively, Rennes, St Jaques, nutrients: Syndicat Mixte du Grand Bassin de l’Oust, SMGBO, Bretagne, France.

## Supplementary Materials

The following are available online at https://www.mdpi.com/article/10.3390/toxins13050351/s1, Figure S1. Plots from the function gam.check () for each abiotic parameters: At the top left, the Q—Q plot compares the model residuals to a normal distribution., Table S1. Microcystin types meas-ured over the 2007–2019 summer periods.
