# Supplementary material for: Is the Cyanobacterial Bloom Composition Shifting Due to Climate Forcing or Nutrient Changes? Example of a Shallow Eutrophic Reservoir
Source: Toxins (Basel). 2021 May 13;13(5):351. doi: 10.3390/toxins13050351 (PMC8153585; doi:10.3390/toxins13050351)
Supplement: Supplementary file 1 [file toxins-13-00351-s001.zip › toxins-1183728-supplementary header changed.pdf]

Supplementary Materials: Is the Cyanobacterial Bloom Composition Shifting due to Climate Forcing or Nutrient Changes? Example of a Shallow Eutrophic Reservoir

Table S1. Microcystin types measured over the 2007–2019 summer periods. Only years with positive concentrations in lake water are shown. For all other years, the values are below the detection limits.

Year	Date	Microcystin type (μg L^{-1})	Total Microcystin (μg L^{-1})	MC-LR toxicity equivalents (μg L^{-1})
2013	2/9	LR (1.2) YR (0.6) RR (0.3)	2.1	1.83
2015	30/6	LA (0.5)	0.5	0.5
	15/7	LR (0.6) YR (0.3	0.9	0.9
	20/7	LR (0.5)	0.5	0.5
	28/7	LR (0.3) YR (0.3)	0.6	0.6
	18/8	LA (0.3)	0.3	0.3
2018	16/7	LR (0.5)	0.5	0.5
	23/7	LR (0.3)	0.3	0.3
	20/8	LR (0.3)	0.3	0.3

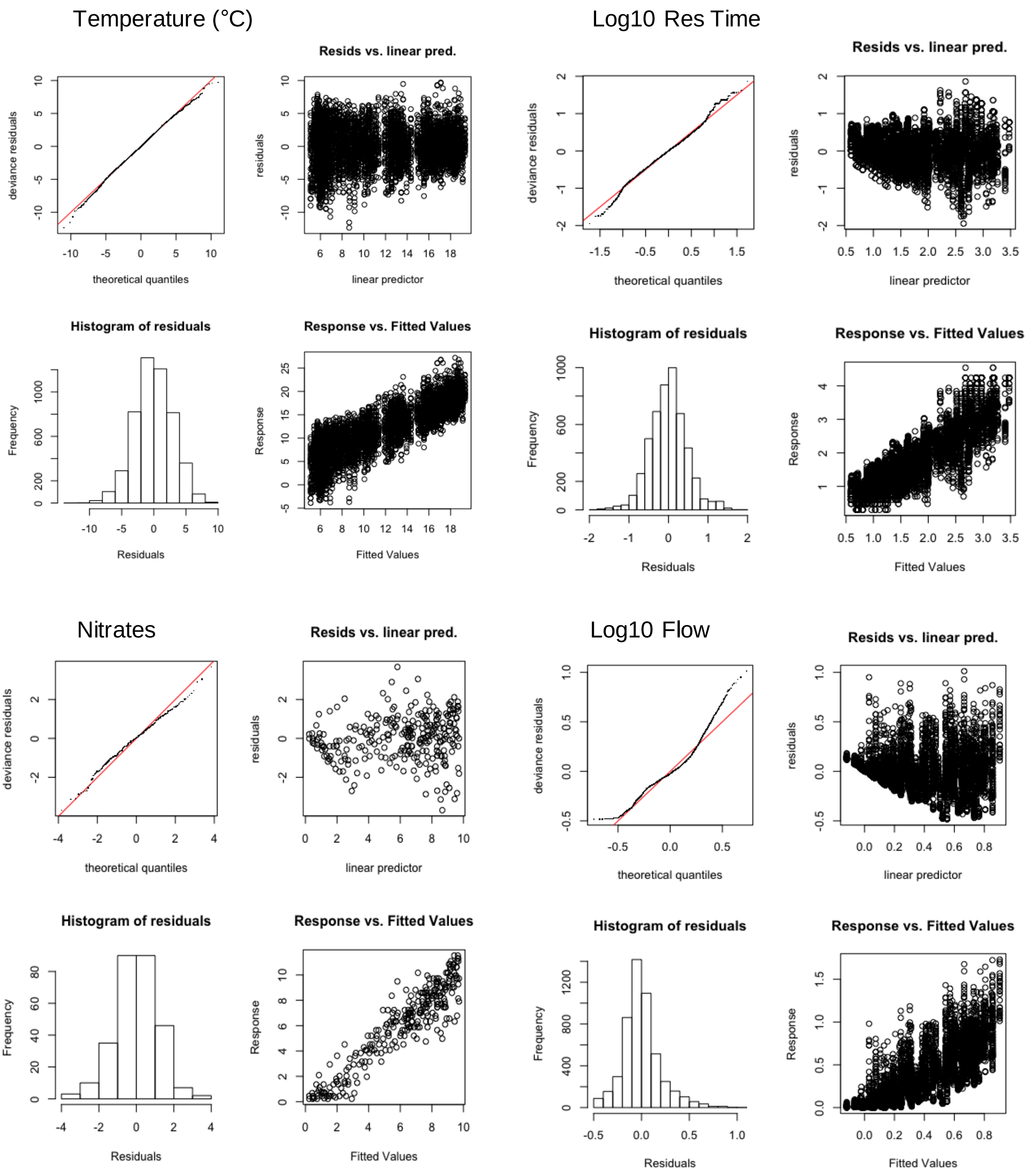

Figure S1. Plots from the function gam.check () for each abiotic parameters: At the top left, the Q—Q plot compares the model residuals to a normal distribution. At the bottom left, the histogram of the residuals is shown. At the top right, residual values depending on the predicted values are plotted. At the bottom right, the observed values are plotted depending on fitted values.
